# Autism Diagnosis Among US Children and Adults, 2011-2022

**DOI:** 10.1001/jamanetworkopen.2024.42218

**Published:** 2024-10-30

**Authors:** Luke P. Grosvenor, Lisa A. Croen, Frances L. Lynch, Ben J. Marafino, Melissa Maye, Robert B. Penfold, Gregory E. Simon, Jennifer L. Ames

**Affiliations:** 1 Division of Research, Kaiser Permanente Northern California, Pleasanton, California; 2 Kaiser Permanente Bernard J. Tyson School of Medicine, Pasadena, California; 3 Center for Health Research, Kaiser Permanente, Portland, Oregon; 4 Center for Health Services Research, Henry Ford Health System, Detroit, Michigan; 5 Pediatrics, College of Human Medicine, Michigan State University, East Lansing; 6 Kaiser Permanente Washington Health Research Institute, Seattle, Washington

## Abstract

**Importance:**

An improved understanding of autism spectrum disorder (ASD) prevalence over time and across the lifespan can inform health care service delivery for the growing population of autistic children and adults.

**Objective:**

To describe trends in the prevalence of ASD diagnoses using electronic records data from a large network of health systems in the US.

**Design, Setting, and Participants:**

This cross-sectional study examined annual diagnosis rates in health records of patients in US health systems from January 1, 2011, to December 31, 2022. Eligible individuals were included in the study sample for a given calendar year if they were enrolled in a participating health system for at least 10 months out of the year. Data were extracted from 12 sites participating in the Mental Health Research Network, a consortium of research centers embedded within large, diverse health care systems.

**Main Outcome and Measures:**

Diagnoses of ASD were ascertained using *International Classification of Diseases, Ninth Revision (ICD-9)* and *International Statistical Classification of Diseases and Related Health Problems, Tenth Revision (ICD-10)* revision codes. Annual diagnosis rates were calculated as the number of unique members diagnosed, divided by the total members enrolled.

**Results:**

A total of 12 264 003 members were enrolled in 2022 (2 359 359 children aged 0 to 17 years [19.2%]; 6 400 222 female [52.2%]; 93 002 American Indian or Alaska Native [0.8%], 1 711 950 Asian [14.0%], 952 287 Black or African American [7.8%], 2 971 355 Hispanic [24.2%], 166 144 Native Hawaiian or Pacific Islander [1.4%], and 6 462 298 White [52.7%]). The ASD diagnosis rate was greatest among 5-to-8-year-olds throughout the study period and increased by 175% among the full sample, from 2.3 per 1000 in 2011 to 6.3 per 1000 in 2022. The greatest relative increase in diagnosis rate from 2011 to 2022 occurred among 26-to-34-year-olds (450%) and increases were greater for female vs male individuals among children (305% [estimated annual percentage change (EAPC), 13.62 percentage points; 95% CI, 12.49-14.75 percentage points] vs 185% [EAPC, 9.63 percentage points; 95% CI, 8.54-10.72 percentage points], respectively) and adults (315% [EAPC, 13.73 percentage points; 95% CI, 12.61-14.86 percentage points] vs 215% [EAPC, 10.33 percentage points; 95% CI, 9.24-11.43 percentage points]). Relative increases were greater in racial and ethnic minority groups compared with White individuals among children, but not adults.

**Conclusions and Relevance:**

In this cross-sectional study of children and adults in the US, ASD diagnosis rates increased substantially between 2011 and 2022, particularly among young adults, female children and adults, and children from some racial or ethnic minority groups. Diagnosis prevalence trends generated using health system data can inform the allocation of resources to meet the service needs of this growing, medically complex population.

## Key Points

**Question:**

How have autism diagnosis rates changed over time among children and adults seeking care from a network of health systems in the US?

**Findings:**

In this cross-sectional study of electronic US health and insurance claims records for over 9 million individuals per year from 2011 to 2022, relative increases in autism diagnosis rates were greatest among young adults compared with all other age groups, female compared with male individuals, and some racial and ethnic minority groups compared with White individuals among children but not adults.

**Meaning:**

Patterns of increase in autism diagnosis rates reflect a need for expanded health care services and continued research on sociodemographic disparities among this growing population.

## Teaser

This cross-sectional study examines relative increases in autism diagnosis rates in US health and insurance claims records from 2011 to 2022 by age, gender, race, and ethnicity.

## Introduction

Autism spectrum disorder (ASD) is a neurodevelopmental condition diagnosed by the presence of social communication impairments and restricted and repetitive behaviors.^1^ The lifelong presentation of ASD often co-occurs with multiple physical and mental health conditions^2^ leading to diverse health service utilization and high annual medical costs.^3^ Prevalence among children in the US has risen over 4-fold in the past 2 decades, from 6.7 cases per 1000 (1 in 150) in 2000^4^ to 27.6 per 1000 (1 in 36) in 2020.^5^

Hypothesized reasons for prevalence increases include changes to developmental screening practices,^6,7^ diagnosis definitions,^8^ policies, and environmental factors as well as increased advocacy and education.^9^ Prevalence estimates can also vary by case ascertainment methods^10^ or geographic region. For example, the most recent Centers for Disease Control and Prevention (CDC) Autism and Developmental Disabilities Monitoring (ADDM) Network estimates ranged from 23.1 per 1000 children in Maryland to 44.9 per 1000 in California.^5^

Male individuals are about 4 times as likely as females to be diagnosed with ASD.^5^ Sources of prevalence discrepancies include sex differences in genetic predisposition^11,12,13,14^ and sex- or gender-specific phenotypic presentations leading to delayed or missed diagnoses in females.^15,16^ Sociocultural perceptions and gender behavior norms can also cause female individuals to socially hide autistic traits (commonly referred to as “camouflaging”),^17^ contributing to underdiagnosis.^18^ Improved understanding of sex- or gender-related prevalence differences may inform strategies for early identification and intervention.

Prior to 2016, ASD prevalence was consistently higher among White children than other racial or ethnic groups.^5,19^ Relative increases in prevalence have since been greater among Black, Hispanic, and Asian or Pacific Islander children; in 2020, prevalence was lowest among White children for the first time.^5^ While regional variability in ASD prevalence indicates persistence of racial or ethnic disparities,^5,20^ national trends reported by the CDC may be reflective of improved identification, outreach, and access to services for historically underserved groups.^5,19^

Although typically diagnosed in early childhood, autism is a lifelong condition that typically requires specialized support into adulthood.^21^ However, in contrast to routine surveillance and reporting of ASD prevalence among school-aged children, few studies have described prevalence among adults. Studies of nationwide Medicaid claims data from 2008 to 2019 reported increases in prevalence that were most pronounced among younger adults ages 18 to 34 years and a stable male-to-female prevalence ratio of approximately 3 to 1.^22,23^ Unlike among children, racial and ethnic disparities seem to have persisted among adults; a 2023 study^23^ reported that White adults were at least twice as likely as other groups to be diagnosed.

As the medically complex population of autistic people in the US continues to grow and age into adulthood, projections of support needs across the lifespan will become increasingly important. Health system data provide a robust, naturalistic setting for characterizing changes in diagnosis rates with potential to directly inform service delivery. The present study examined trends in ASD diagnosis rates related to age, gender, race, and ethnicity using data from children and adults enrolled within integrated health care systems in the US.

## Methods

### Study Sample

Electronic health records and insurance claims data were translated to a common set of standards via a federated virtual data warehouse model^24^ and extracted for individuals seeking care from one of 12 sites of the Mental Health Research Network (MHRN)^25^ (eTable 1 in Supplement 1) between January 1, 2011, and December 31, 2022. Data were included in the study for a given calendar year from individuals enrolled for 10 months or more of that year. Institutional review boards at respective sites approved use of their data and granted waivers of informed consent for the use of deidentified data for this research. We followed the Strengthening the Reporting of Observational Studies in Epidemiology (STROBE) checklist for reporting cross-sectional studies.

### Outcome

Diagnoses of ASD were ascertained using *International Classification of Diseases, Ninth Revision (ICD-9)* or *International Statistical Classification of Diseases and Related Health Problems, Tenth Revision (ICD-10)* codes (eTable 2 in Supplement 1) extracted from member records. For each year in the study period, individuals were counted as diagnosed with ASD by the presence of at least 1 diagnostic code during that year.

### Sociodemographic Characteristics

Age was calculated as the number of complete years between date of birth and January 1 of each study year and categorized as: 0 to 4 years, 5 to 8 years, 9 to 12 years, 13 to 17 years, 18 to 25 years, 26 to 34 years, 35 to 44 years, 45 to 54 years, 55 to 64 years, and 65 years and older. We also stratified the sample into 2 groups, children (ages 0 to 17 years) and adults (ages 18 years and older), to ease interpretation of findings based on known prevalence differences across age.^5,26^ Gender was extracted from enrollment data and coded as male, female, or other (including transgender). Race and ethnicity data represented self-reported (or parent- or guardian-reported) responses to standard categories, typically recorded at outpatient visit registration, and included Asian, Black or African American, Native Hawaiian or other Pacific Islander, American Indian or Alaska Native, White, multiracial, unknown, or other. Hispanic ethnicity categories were yes, no, or unknown.

### Statistical Analysis

For each year between 2011 and 2022, we determined frequencies of autistic enrollees and all enrollees within strata of sociodemographic characteristics. We calculated the annual prevalence of ASD diagnosis (referred to hereafter as *diagnosis rate*) standardized per 1000 persons, as the number of members with a recorded diagnosis divided by the total members enrolled that year and computed 95% binomial proportion confidence intervals using the Wilson score method.^27^ Rates were generated separately for each MHRN site and for all sites combined, as well as stratified by age group, gender, race, and ethnicity. To examine trends over time, we calculated relative changes in diagnosis rates as percentages, comparing 2022 with 2011 to obtain 1 rate of change for the study period and comparing year-over-year rates to examine trends in shorter periods. We modeled changes in diagnosis rates over time using weighted least squares regression with log-transformed diagnosis rate as the outcome and inverse variance of the rate as weights. We then calculated estimated annual percentage change (EAPC) in diagnosis rates and 95% CIs from model coefficients for time to describe trends within strata.^28^ Furthermore, we fitted weighted least squares models with group-by-time interaction terms to test significance of differences in rate change across strata for each group: age-by-time, gender-by-time, race-by-time and ethnicity-by-time. All tests were 2-sided, with *P* < .05 interpreted as statistically significant. Analyses were conducted using R, version 4.3.3 (R Project for Statistical Computing).

## Results

### Study Sample

Among 12 264 003 members enrolled in 2022, 2 359 359 (19.2%) were under 18 years of age and 6 400 222 (52.2%) were female; 93 002 American Indian or Alaska Native (0.8%), 1 711 950 were Asian (14.0%), 952 287 Black or African American (7.8%), 2 971 355 Hispanic (24.2%), 166 144 Native Hawaiian or Pacific Islander (1.4%), and 6 462 298 White (52.7%) (Table). In contrast, 56 553 (72.8%) of the 77 683 autistic enrollees were under age 18 years and 20 019 (25.8%) were female. Distributions of the 2 groups were similar with respect to race and ethnicity: the majority reported being of White race (6 462 298 [52.7%] of the total sample and 41 618 [53.6%] of autistic enrollees) and approximately a quarter of each was Hispanic (2 971 355 [24.2%] of total sample and 21 775 [28.0%] of autistic enrollees), while 2 749 574 (22.4%) and 5 104 721 (41.6%) individuals in the full sample had unknown race or ethnicity, respectively.

**Table.** Sociodemographic Characteristics and Autism Diagnosis Rates for Members at Participating MHRN Sites in 2022

Characteristic	Individuals, No. (%)		Diagnosis rate per 1000 persons (95% CI)
	Diagnosed with ASD (n = 77 683)	Total enrolled (n = 12 263 980)	
Age group, y			
0-4	17 315 (22.3)	601 042 (4.9)	28.8 (28.4-29.2)
5-8	15 211 (19.6)	502 298 (4.1)	30.3 (29.8-30.8)
9-12	11 698 (15.1)	530 393 (4.3)	22.1 (21.7-22.5)
13-17	12 329 (15.9)	725 626 (5.9)	17.0 (16.7-17.3)
18-25	11 185 (14.4)	1 173 625 (9.6)	9.5 (9.4-9.7)
26-34	5504 (7.1)	1 485 262 (12.1)	3.7 (3.6-3.8)
35-44	2276 (2.9)	1 751 364 (14.3)	1.3 (1.2-1.4)
45-54	1006 (1.3)	1 672 683 (13.6)	0.6 (0.6-0.6)
55-64	665 (0.9)	1 704 694 (13.9)	0.4 (0.4-0.4)
≥65	494 (0.6)	2 116 993 (17.3)	0.2 (0.2-0.3)
Gender			
Female	20 019 (25.8)	6 400 222 (52.2)	3.1 (3.1-3.2)
Male	57 636 (74.2)	5 861 914 (47.8)	9.8 (9.8-9.9)
Other	15 (<0.1)	493 (<0.1)	30.4 (18.5-49.6)
Unknown^a^	11 (<0.1)	1282 (<0.1)	8.6 (4.8-15.3)
Race			
American Indian or Alaska Native	757 (1.0)	93 002 (0.8)	8.1 (7.6-8.7)
Asian	10 819 (13.9)	1 711 950 (14.0)	6.3 (6.2-6.4)
Black or African American	6965 (9.0)	952 287 (7.8)	7.3 (7.1-7.5)
Native Hawaiian or other Pacific Islander	980 (1.3)	166 144 (1.4)	5.9 (5.5-6.3)
White	41 618 (53.6)	6 462 298 (52.7)	6.4 (6.4-6.5)
Unknown^a^	15 821 (20.4)	2 749 574 (22.4)	5.8 (5.7-5.8)
Multiracial	72 (0.1)	23 830 (0.2)	3.0 (2.4-8.3)
Other	651 (0.8)	103 728 (0.8)	6.3 (5.8-6.8)
Hispanic ethnicity			
Yes	21 775 (28.0)	2 971 355 (24.2)	7.3 (7.2-7.4)
No	25 263 (32.5)	4 187 927 (34.1)	6.0 (6.0-6.1)
Unknown	30 645 (39.4)	5 104 721 (41.6)	6.0 (5.9-6.1)

Abbreviations: ASD, autism spectrum disorder; MHRN, Mental Health Research Network.

^a^ Gender, race, or ethnicity may have been missing or not recorded, and categorized as unknown, because a member or parent (or guardian) missed a visit, was not asked, or declined to respond. Other includes people who selected other as well as those who selected a category available at only 1 or a subset of sites, which was then recategorized to other as part of data harmonization prior to extraction.

### Overall Trends

Prevalence of ASD diagnosis in 2022 was highest among 5-to-8-year-olds (30.3 per 1000 children) and declined with age, particularly among those aged 45 years or older (ages 45-54 years: 0.6 per 1000 persons; 55-64 years: 0.4 per 1000 persons; ages 65 years or older: 0.2 per 1000 persons) (Table). The 2022 diagnosis rate was higher among males than females at an approximately 3 to 1 ratio (9.8 per 1000 males vs 3.1 per 1000 females). By race and ethnicity, prevalence was highest among Native individuals (8.1 per 1000 persons), lowest among multiracial members (3.0 per 1000 persons) and was higher among Hispanic than non-Hispanic enrollees (7.3 per 1000 persons vs 6.0 per 1000 persons, respectively).

**Figure 1.**
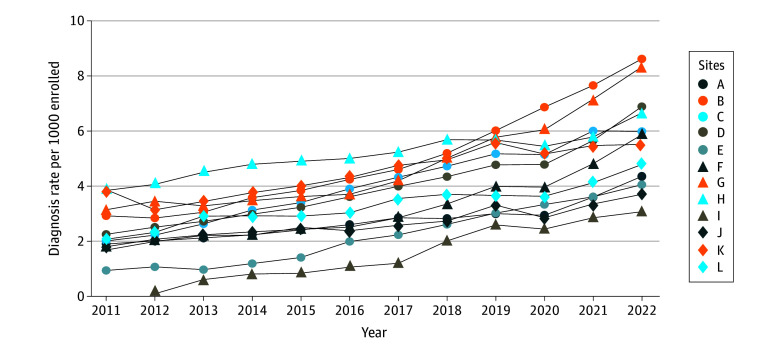
Annual Autism Diagnosis Rates Among Members at 12 MHRN Sites From 2011 to 2022 MHRN indicates Mental Health Research Network.

From 2011 to 2022, ASD diagnosis rate among all enrollees increased by 175% (from 2.3 per 1000 persons to 6.3 per 1000 persons; EAPC, 9.41 percentage points [95% CI, 8.32-10.51 percentage points], *P* < .001) (eTable 3 in Supplement 1) and relative increases at each MHRN site ranged from 42% to 333% (Figure 1; eTable 4 in Supplement 1). Diagnosis rates increased steadily from 2011 to 2019, with year-to-year relative increases ranging from 7.2% to 11.2%. Prevalence increased by just 0.6% from 2019 to 2020, and then increased by 16.5% from 2020 to 2021 and 11.9% from 2021 to 2022. The number of autistic enrollees increased at a disproportionately lower rate than the number of total enrollees from 2019 to 2020 (2.4% and 1.8%, respectively) compared with all other 2-year periods (eg, from 2017 to 2018: 13.2% and 3.3%, respectively) (eTable 3 in Supplement 1).

### Trends by Gender

**Figure 2.**
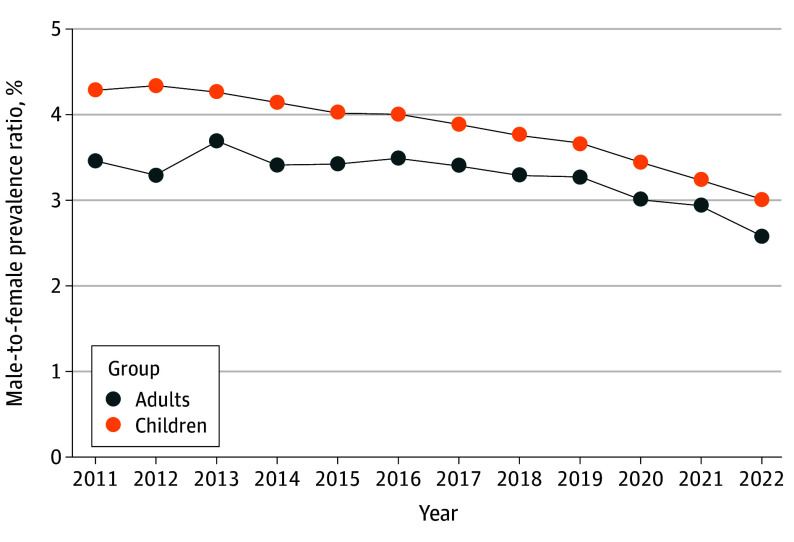
Male-to-Female Autism Prevalence Ratio Among Children and Adults From 2011 to 2022

Annual diagnosis rates were higher among male than female individuals within each study year for both children and adults (eFigure 1 in Supplement 1). Rates increased significantly from 2011 to 2022 for both genders, and relative increases were greater among female than male children (female: 305% increase; EAPC, 13.62 [95% CI, 12.49-14.75], *P* < .001; male: 185% increase; EAPC, 9.63 percentage points [95% CI, 8.54-10.72 percentage points], *P* < .001; gender-by-time interaction *P* < .001) and adults (female: 315% increase; EAPC, 13.73 percentage points [95% CI, 12.61-14.86 percentage points], *P* < .001; male: 215% increase; EAPC, 10.33 percentage points [95% CI, 9.24-11.43 percentage points], *P* < .001; gender-by-time interaction *P* = .03) (eTable 5 in Supplement 1). The male-to-female prevalence ratio was higher among children than adults for each year and declined for both groups from 2011 to 2022 (children, 4.29:1 to 3.01:1; adults, 3.45:1 to 2.60:1) (Figure 2; eTable 6 in Supplement 1).

### Trends by Age Group

**Figure 3.**
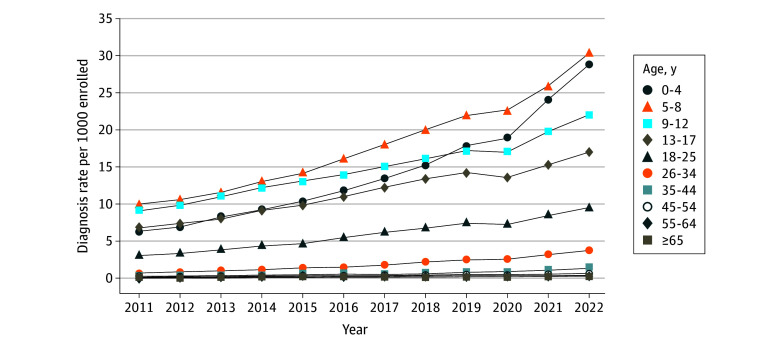
Annual Autism Diagnosis Rates Among Members at MHRN Sites From 2011 to 2022, Stratified by Age Group MHRN indicates Mental Health Research Network.

Annual diagnosis rates were highest among younger age groups and lowest among older groups, and rates increased from 2011 to 2022 for all age groups (Figure 3; eTable 7 in Supplement 1). Relative increases in diagnosis rates from 2011 to 2022 were generally greater among older groups (eg, ages 26 to 34 years: 452%; EAPC, 16.07 percentage points [95% CI, 14.91-17.21 percentage points], *P* < .001; ages 35 to 44 years, 338%; EAPC, 15.13 percentage points [95% CI, 14.00-16.27 percentage points]) compared with younger (eg, ages 5 to 8 years: 207%; EAPC, 10.62 percentage points [95% CI, 9.51-11.72 percentage points], *P* < .001; ages 9 to 12 years: 143%; EAPC, 7.73 percentage points [95% CI, 6.66-8.80 percentage points], *P* < .001). The exception was among 0-to-4-year-olds, with prevalence increasing by 352% (from 6.4 per 1000 persons in 2011 to 28.8 per 1000 persons in 2022; EAPC, 15.19 percentage points [95% CI, 14.05-16.33 percentage points], *P* < .001; age-by-time interaction *P* < .001) (Figure 3; eTable 7 in Supplement 1).

### Trends by Race

**Figure 4.**
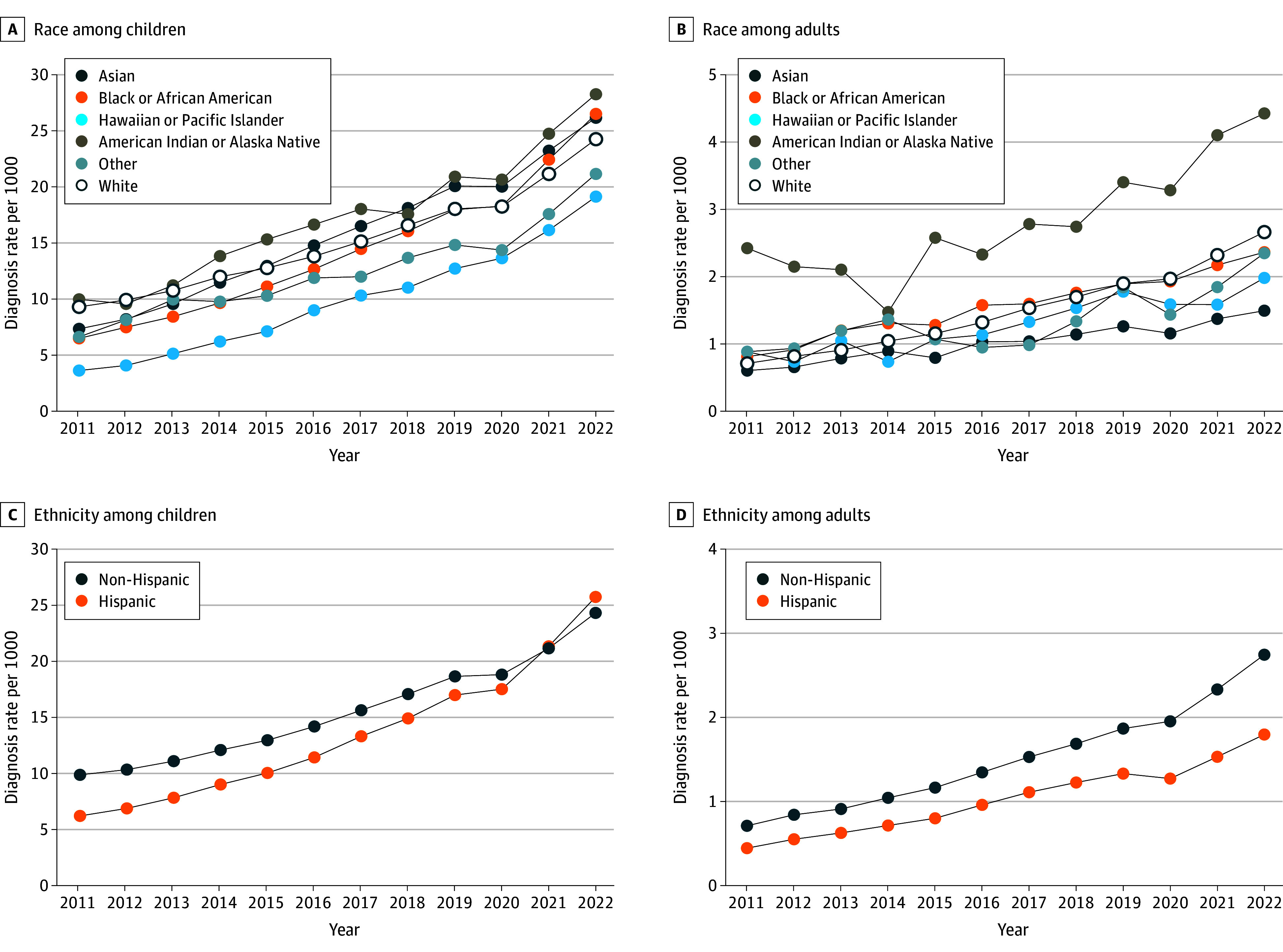
Annual Autism Diagnosis Rates Among Members at MHRN Sites From 2011 to 2022, Stratified by Reported Race and Ethnicity MHRN indicates Mental Health Research Network.

Prevalence also increased significantly over time within each racial group, ranging from 61% among American Indian or Alaska Native (from 6.5 per 1000 persons in 2011 to 10.5 per 1000 persons in 2022; EAPC, 5.43 percentage points [95% CI, 4.39-6.47 percentage points], *P* < .001) to 205% among Hawaiian Pacific enrollees in the full sample (from 2.4 per 1000 persons to 7.3 per 1000 persons; EAPC, 9.60 percentage points [95% CI, 8.52-10.69 percentage points], *P* < .001) (eTable 8 in Supplement 1). These trends varied for children and adults; for example, increases among children were greatest within the Hawaiian Pacific (428%, from 3.6 per 1000 persons to 19.1 per 1000 persons; EAPC, 13.95 percentage points [95% CI, 12.82-15.08 percentage points], *P* < .001) and Black or African American (EAPC, 13.10 percentage points [95% CI, 11.99-14.22 percentage points], *P* < .001) groups, and these increases were significantly greater than among White children (race-by-time interaction *P* = .006 and *P* = .05, respectively) (Figure 4A). The greatest increase among adults occurred within White individuals (275%, from 0.7 per 1000 persons to 2.7 per 1000 persons; EAPC, 12.29 percentage points [95% CI, 11.19-13.40 percentage points], *P* < .001). While increases appeared considerably greater than among other race groups, these differences were not statistically significant (for example, compared with American Indian or Alaska Natives: 83% increase, from 2.4 per 1000 persons to 4.4 per 1000 persons; EAPC, 8.36 percentage points [95% CI, 7.29-9.43 percentage points], *P* < .001; race-by-time interaction *P* = .08) (Figure 4B).

### Trends by Ethnicity

Increases in diagnosis rates from 2011 to 2022 were significantly greater among Hispanic compared with non-Hispanic children (Hispanic: 315% increase, from 6.2 per 1000 persons to 25.7 per 1000 persons; EAPC, 13.39 percentage points [95% CI, 12.26-14.50 percentage points], *P* < .001; non-Hispanic: 146%, from 9.9 to 24.3 per 1000 persons; EAPC, 8.50 percentage points [95% CI, 7.43-9.57 percentage points], *P* < .001; ethnicity-by-time interaction *P* < .001) (Figure 4C; eTable 9 in Supplement 1). Increases were significant among both groups of adults but did not differ across ethnicity over time (Hispanic: 303% increase, from 0.4 per 1000 persons to 1.8 per 1000 persons; EAPC, 11.89 percentage points [95% CI, 10.78-13.00 percentage points], *P* < .001; non-Hispanic: 286%, from 0.7 per 1000 persons to 2.7 per 1000 persons; EAPC, 12.45 percentage points [95% CI, 11.33-13.55 percentage points]; ethnicity-by-time interaction *P* = .52) (Figure 4D). Stratifying by both ethnicity and age group revealed that relative increases were greatest among Hispanic children ages 0 to 4 and 5 to 8 years. For example, diagnosis rates in 2011 were lower among Hispanic compared with non-Hispanic 5-to-8-year-olds (8.1 per 1000 persons and 12.9 per 1000 persons, respectively) and by 2022, rates were instead higher among Hispanic than non-Hispanic children of this age (34.5 per 1000 persons vs 30.2 per 1000 persons, respectively) (eFigure 2 in Supplement 1). Differences across strata of ethnicity and race should be interpreted with caution considering the high percentage of participants who reported unknown for these variables. Results from linear models testing time trends within strata of all sociodemographic variables are presented in eTable 10 in Supplement 1.

## Discussion

We identified trends and disparities in ASD diagnosis rates among a large, diverse population of children and adults in the US. Rates for each year from 2011 to 2022 were highest among young children, whereas relative increases were greatest for young adults. While annual diagnosis rates were consistently higher among males, the relative increase from 2011 to 2022 was significantly greater for females, among both children and adults. Across racial and ethnic group strata, annual diagnosis rates tended to be highest among American Indian or Alaska Native children and adults. Relative increases were greater within some racial and ethnic minority groups compared with White members among children, but not adults.

Our findings should be considered in the context of past studies of ASD prevalence including CDC ADDM Network reports of substantial increases among school-age children.^5,20,29^ We found increases in diagnosis rates over time across strata of age, gender, race, and ethnicity among both children and adults. Annual rates were highest among the youngest children, as expected based on improved early detection including universal pediatric developmental screening.^5,30^ Diagnosis rate increases among adults were greatest within 18-to-25 and 26-to-34-year-olds, consistent with recent findings from adult Medicaid claims data over similar periods.^22,23^ Rates reported here may underestimate the true prevalence of ASD in adults, especially older female adults, as many would not have been screened in childhood and remain undiagnosed.^31^ Nevertheless, our findings indicate that the population of autistic adults in the US will continue to grow, underscoring a need for expanded health care services.

The male-to-female prevalence ratio among children and adults steadily declined from 2011 to 2022. While this contrasts with a generally accepted notion that the 4:1 male-to-female prevalence ratio in ASD has remained stable, our findings are consistent with CDC data showing an almost 20% decrease among children, from 4.5:1 in 2012^32^ to 3.8:1 in 2020.^5^ Relative increases in diagnosis rates were significantly higher among female than male adults, which is in line with a 2024 report of substantial decreases in the male-to-female ratio among adults in North Carolina, from 5.6:1 in 2000 to 3.1:1 in 2021.^33^ Increased awareness of ASD presentation in females, for example related to expanded representation in social media^34^ or improved provider tools^35^ and training programs,^36^ is one potential factor underlying these changes. Although we could not determine age at diagnosis, multiple studies have reported increases in new diagnoses among female adults.^10,33^

Relative increases in prevalence were greater among Black, Asian, American Indian or Alaska Native, and Hispanic children compared with White children. Our findings are consistent with CDC ADDM Network findings from 2000 to 2020^5,19^ and a 2022 study of health records from 2017 to 2021 that additionally reported comparable median ages at diagnosis across racial and ethnic groups.^37^ Attenuation of disparities over time could reflect improved detection within MHRN sites related to implementation of universal screening and increased outreach to minority communities. While this may represent a shift away from racial and ethnic disparities in diagnostic practices,^5,19^ there is evidence that disparities persist in both educational and service delivery settings, including primary care.^38,39,40^

Unlike among children, racial and ethnic disparities persisted over time among adults and ASD diagnosis rates were greater among White compared with Hispanic and Black adults. These findings align with recent Medicaid studies, such as Rubenstein et al,^23^ and may represent the lasting impact of historically inequitable screening and diagnosis practices, as well as existing barriers to care faced by autistic adults.^41,42^ Future research should continue to monitor disparities, especially as they relate to improved diagnostic tools for adults^43^ or policy changes (eg, state mandate age caps^44^). We also found that annual prevalence was highest throughout the study period among American Indian or Alaska Native children and adults. This difference has not been reported by past studies of adults, although the CDC ADDM Network reported similar findings in 2018 for American Indian or Alaska Native children.^20^ Higher diagnosis rates in our data may result from proximity or partnerships between Native communities and specific MHRN sites, including preferential selection into health systems because of insurance coverage type and/or availability of ASD-specific services. Additionally, a higher prevalence of mental and physical disabilities has been reported among American Indian or Alaska Natives.^45^

Existing disparities in prevalence rates underscore a need for expanded diagnostic and specialty health care services particularly for adolescents and young adults, among whom relative increases in diagnosis rates were highest. This population faces significant challenges to addressing health care needs, also referred to as the “services cliff,” in part due to lack of comprehensive or integrated services and accommodations in adult care.^46^ Continuity of care is especially important considering autistic young adults experience elevated rates of diabetes, obesity, anxiety, depression, and other conditions.^2^ A detailed understanding of changes in diagnosis rates among specific age, gender, and racial and ethnic groups may enable clinical providers to better meet the needs of this growing and medically complex population.

After consistent year-to-year increases in diagnosis rate from 2011 to 2019, there were no increases in 2020, likely because of widespread disruptions to diagnostic services caused by the COVID-19 pandemic.^47^ Autistic people and their families were less likely than others to access health services during this time,^48^ despite efforts by diagnostic centers to adjust practices by implementing telehealth evaluations.^49^ Diagnosis rates again increased after 2020 and were higher in 2021 and 2022 than prior years. Further research is warranted as to whether this reflects increased access to diagnostic services versus any true increase in diagnosis prevalence.

### Limitations

This study had several limitations. Our research demonstrates the utility of administrative health data as a naturalistic setting for studying trends in ASD diagnosis rates. Our use of 1 or more ASD diagnoses for case identification is less stringent than surveillance or etiologic studies, but sufficient for describing diagnosis trends over time and consistent with similar studies.^22,23^ Additionally, if ASD was not recorded for health system encounters unrelated to ASD, then diagnoses rates may have been underestimated, particularly among adults. While our large sample was sociodemographically and geographically diverse, generalizability may be limited by use of data from only integrated health systems. For example, we could not capture the experiences of persons without insurance coverage or who sought ASD-related services from other settings. High rates of missing or unknown data reported by some sites for race and ethnicity may have further limited generalizability and could have affected the accuracy of or introduced biases in diagnosis rates, particularly among smaller groups. While the transition from *ICD-9* to *ICD-10* codes in 2015 was previously found to have little to no impact on ASD diagnosis rates,^50^ differences in diagnostic practices between or within MHRN sites may have influenced trends. Lastly, we were unable to examine trends by potentially important variables including insurance provider type, age at diagnosis or co-occurring intellectual disability. Future work should characterize prevalence trends by these and other factors to represent individuals with varying levels of service needs and presentations of ASD.^51^

## Conclusions

We identified trends and disparities in autism diagnosis rates in the US from 2011 to 2022, and found the greatest relative increases among young adults, females, and children in multiple racial and ethnic minority groups. These findings forecast a substantial number of autistic people aging into adult care and can be used both to inform interventions for addressing disparities and to efficiently allocate resources to meet the support needs of autistic people across the lifespan.

## Notes

Supplement 1. **eTable 1.** Participating Sites of the Mental Health Research Network From Which Data Were Extracted for This Study **eTable 2.** *International Classification of Diseases, Ninth Revision* and *Tenth* Revision Codes Used to Identify Autism Within Administrative Health Records at All MHRN Sites **eTable 3.** Summary of Enrollment Information and ASD Diagnosis Rates for the Full Study Samples Within Each Year From 2011 to 2022 **eTable 4.** Count of Autistic Individuals and Total Individuals Enrolled at Each Study Site and in Each Study Year From 2011-2022, with ASD Diagnosis Rate per 1000 and 95% Confidence Intervals **eTable 5.** Count of Autistic Individuals and Total Individuals Enrolled for Female and Male Gender and Each Study Year, With Diagnosis Rate per 1000 and 95% Confidence Intervals **eTable 6.** Male-to-Female ASD Prevalence Ratios for Each Study Year in 2011-2022, for the Full Study Sample and Separately for Children (Ages 0-17) and Adults (Ages 18+) **eTable 7.** Count of Autistic Individuals and Total Individuals Enrolled Each Age Group and Each Study Year, With ASD Diagnosis Rates per 1000 and 95% Confidence Intervals **eTable 8.** Count of Autistic Individuals and Total Individuals Enrolled for Children and Adults With Diagnosis Rate per 1000 and 95% Confidence Intervals, Stratified by Race Group for Each Year in 2011-2022 **eTable 9.** Count of Autistic Individuals and Total Individuals Enrolled With ASD Diagnosis Rate per 1000 and 95% Confidence Intervals for Children and Adults in Each Ethnicity Group (Hispanic, Non-Hispanic) and Each Study Year in 2011-2022 **eTable 10.** Results From Weighted Least Squares Models Testing Significance of Changes in Diagnosis Rates From 2011-2022 Within Strata of Age, Gender, Race, and Ethnicity **eFigure 1.** Prevalence of ASD Diagnosis per 1000 Enrollees Among Children and Adults Enrolled at All MHRN Sites From 2011-2022, Stratified by Reported Gender **eFigure 2.** Annual Prevalence of ASD Diagnosis per 1000 Enrollees in 2011 and 2022, Stratified by Age Group and Hispanic Ethnicity

Supplement 2.
Data Sharing Statement
