# Supplementary material for: Autism Diagnosis Among US Children and Adults, 2011-2022
Source: JAMA Netw Open. 2024 Oct 30;7(10):e2442218. doi: 10.1001/jamanetworkopen.2024.42218 (PMC11525601; doi:10.1001/jamanetworkopen.2024.42218)
Supplement: Supplement 1. — eTable 1. Participating Sites of the Mental Health Research Network From Which Data Were Extracted for This Study eTable 2. International Classification of Diseases, Ninth Revision and Tenth Revision Codes Used to Identify Autism Within Administrative Health Records at All MHRN Sites eTable 3. Summary of Enrollment Information and ASD Diagnosis Rates for the Full Study Samples Within Each Year From 2011 to 2022 eTable 4. Count of Autistic Individuals and Total Individuals Enrolled at Each Study Site and in Each Study Year From 2011-2022, with ASD Diagnosis Rate per 1000 and 95% Confidence Intervals eTable 5. Count of Autistic Individuals and Total Individuals Enrolled for Female and Male Gender and Each Study Year, With Diagnosis Rate per 1000 and 95% Confidence Intervals eTable 6. Male-to-Female ASD Prevalence Ratios for Each Study Year in 2011-2022, for the Full Study Sample and Separately for Children (Ages 0-17) and Adults (Ages 18+) eTable 7. Count of Autistic Individuals and Total Individuals Enrolled Each Age Group and Each Study Year, With ASD Diagnosis Rates per 1000 and 95% Confidence Intervals eTable 8. Count of Autistic Individuals and Total Individuals Enrolled for Children and Adults With Diagnosis Rate per 1000 and 95% Confidence Intervals, Stratified by Race Group for Each Year in 2011-2022 eTable 9. Count of Autistic Individuals and Total Individuals Enrolled With ASD Diagnosis Rate per 1000 and 95% Confidence Intervals for Children and Adults in Each Ethnicity Group (Hispanic, Non-Hispanic) and Each Study Year in 2011-2022 eTable 10. Results From Weighted Least Squares Models Testing Significance of Changes in Diagnosis Rates From 2011-2022 Within Strata of Age, Gender, Race, and Ethnicity eFigure 1. Prevalence of ASD Diagnosis per 1000 Enrollees Among Children and Adults Enrolled at All MHRN Sites From 2011-2022, Stratified by Reported Gender eFigure 2. Annual Prevalence of ASD Diagnosis per 1000 Enrollees in 2011 and 2022, Stratified by Age Group [file jamanetwopen-e2442218-s001.pdf]

Supplemental Online Content

Grosvenor LP, Croen LA, Lynch FL, et al. Trends in autism diagnosis among US children and adults, 2011-2022. *JAMA Netw Open.* 2024;7(10):e2442218. doi:10.1001/jamanetworkopen.2024.42218

eTable 1. Participating Sites of the Mental Health Research Network From Which Data Were Extracted for This Study

eTable 2. *International Classification of Diseases, Ninth Revision* and *Tenth* Revision Codes Used to Identify Autism Within Administrative Health Records at All MHRN Sites

eTable 3. Summary of Enrollment Information and ASD Diagnosis Rates for the Full Study Samples Within Each Year From 2011 to 2022

eTable 4. Count of Autistic Individuals and Total Individuals Enrolled at Each Study Site and in Each Study Year From 2011-2022, with ASD Diagnosis Rate per 1000 and 95% Confidence Intervals

eTable 5. Count of Autistic Individuals and Total Individuals Enrolled for Female and Male Gender and Each Study Year, With Diagnosis Rate per 1000 and 95% Confidence Intervals

eTable 6. Male-to-Female ASD Prevalence Ratios for Each Study Year in 2011-2022, for the Full Study Sample and Separately for Children (Ages 0-17) and Adults (Ages 18+)

eTable 7. Count of Autistic Individuals and Total Individuals Enrolled Each Age Group and Each Study Year, With ASD Diagnosis Rates per 1000 and 95% Confidence Intervals

eTable 8. Count of Autistic Individuals and Total Individuals Enrolled for Children and Adults With Diagnosis Rate per 1000 and 95% Confidence Intervals, Stratified by Race Group for Each Year in 2011-2022

eTable 9. Count of Autistic Individuals and Total Individuals Enrolled With ASD Diagnosis Rate per 1000 and 95% Confidence Intervals for Children and Adults in Each Ethnicity Group (Hispanic, Non-Hispanic) and Each Study Year in 2011-2022

eTable 10. Results From Weighted Least Squares Models Testing Significance of Changes in Diagnosis Rates From 2011-2022 Within Strata of Age, Gender, Race, and Ethnicity

© 2024 Grosvenor LP et al. *JAMA Network Open.*

eFigure 1. Prevalence of ASD Diagnosis per 1000 Enrollees Among Children and Adults Enrolled at All MHRN Sites From 2011-2022, Stratified by Reported Gender

eFigure 2. Annual Prevalence of ASD Diagnosis per 1000 Enrollees in 2011 and 2022, Stratified by Age Group and Hispanic Ethnicity

This supplemental material has been provided by the authors to give readers additional information about their work.

© 2024 Grosvenor LP et al. *JAMA Network Open.*

eTable 1. Participating sites of the Mental health research Network from which data were extracted for this study.

**Site (region)**
Palo Alto Medical Foundation Research Institute (Northern California)
Harvard Pilgrim Health Care (Massachusetts)
Henry Ford Health System (Michigan)
Essentia Institute of Rural Health (Minnesota)
HealthPartners (Minnesota)
Kaiser Permanente (Colorado)
Kaiser Permanente (Georgia)
Kaiser Permanente (Hawaii)
Kaiser Permanente (Northern California
Kaiser Permanente (Southern California)
Kaiser Permanente (Washington)
Kaiser Permanente (Northwest (Oregon/Southwest Washington)

© 2024 Grosvenor LP et al. *JAMA Network Open.*

eTable 2. International Classification of Diseases, Ninth Revision and Tenth Revision Codes used to identify autism within administrative health records at all MHRN sites.

Revision	Code(s)	Description
**ICD-9**	299.0, 299.00, 299.01	Autistic disorder
**ICD-9**	299.1, 299.10, 299.11	Childhood disintegrative disorder
**ICD-9**	299.8, 299.80, 299.81	Other specified pervasive developmental disorder
**ICD-9**	299.9, 299.90, 299.91	Unspecified pervasive developmental disorder
**ICD-10**	F84.0	Autistic disorder
**ICD-10**	F84.3	Childhood disintegrative disorder
**ICD-10**	F84.5	Asperger's syndrome
**ICD-10**	F84.8	Other pervasive developmental delay
**ICD-10**	F84.9	Pervasive developmental disorder, unspecified

© 2024 Grosvenor LP et al. *JAMA Network Open.*

eTable 3. Summary of enrollment information and ASD diagnosis rates for the full study samples within each year from 2011 to 2022.

Year	Autistic Individuals	Total Enrolled	Diagnosis Rate per 1,000 (95% CI)
2011	21768	9443114	2.3 (2.3-2.3)
2012	24521	9834176	2.5 (2.5-2.5)
2013	27880	10094612	2.8 (2.7-2.8)
2014	32084	10446739	3.1 (3.0-3.1)
2015	36517	11089885	3.3 (3.3-3.3)
2016	42163	11622178	3.6 (3.6-3.7)
2017	47867	11967350	4.0 (4.0-4.0)
2018	54205	12358054	4.4 (4.3-4.4)
2019	59351	12288847	4.8 (4.8-4.9)
2020	60758	12510701	4.9 (4.8-4.9)
2021	70188	12401566	5.7 (5.6-5.7)
2022	77683	12264004	6.3 (6.3-6.4)

© 2024 Grosvenor LP et al. *JAMA Network Open.*

eTable 4. Count of autistic individuals and total individuals enrolled at each study site and in each study year from 2011-2022, with ASD diagnosis rate per 1,000 and 95% confidence intervals.

Site	Year	Autistic Individuals	Total Enrolled	Diagnosis Rate per 1000 (95% CI)
A	2011	995	524969	1.9 (1.8-2.0)
B	2011	1247	427277	2.9 (2.8-3.1)
C	2011	5969	2974018	2.0 (2.0-2.1)
D	2011	7080	3150488	2.2 (2.2-2.3)
E	2011	182	194472	0.9 (0.8-1.1)
F	2011	878	487893	1.8 (1.7-1.9)
G	2011	1877	598074	3.1 (3.0-3.3)
H	2011	2357	613150	3.8 (3.7-4.0)
J	2011	367	219070	1.7 (1.5-1.9)
K	2011	626	161848	3.9 (3.6-4.2)
L	2011	190	91855	2.1 (1.8-2.4)
A	2012	1036	515125	2.0 (1.9-2.1)
B	2012	1220	429468	2.8 (2.7-3.0)
C	2012	6745	3042059	2.2 (2.2-2.3)
D	2012	8153	3244560	2.5 (2.5-2.6)
E	2012	205	191768	1.1 (0.9-1.2)
F	2012	1016	491448	2.1 (1.9-2.2)
G	2012	2168	628432	3.4 (3.3-3.6)
H	2012	2436	599604	4.1 (3.9-4.2)
I	2012	5	89039	0.1 (0.0-0.1)
J	2012	426	214579	2.0 (1.8-2.2)
K	2012	808	257350	3.1 (2.9-3.4)
L	2012	303	130744	2.3 (2.1-2.6)
A	2013	1040	489633	2.1 (2.0-2.3)
B	2013	1312	430544	3.0 (2.9-3.2)
C	2013	8088	3077891	2.6 (2.6-2.7)
D	2013	9016	3310160	2.7 (2.7-2.8)
E	2013	184	189626	1.0 (0.8-1.1)
F	2013	1100	492820	2.2 (2.1-2.4)
G	2013	2012	612810	3.3 (3.1-3.4)
H	2013	2853	634335	4.5 (4.3-4.7)
I	2013	58	94532	0.6 (0.5-0.8)
J	2013	482	218114	2.2 (2.0-2.4)
K	2013	990	287717	3.4 (3.2-3.7)
L	2013	745	256430	2.9 (2.7-3.1)
A	2014	1128	506739	2.2 (2.1-2.4)
B	2014	1606	448046	3.6 (3.4-3.8)
C	2014	9988	3193647	3.1 (3.1-3.2)
D	2014	10114	3389966	3.0 (2.9-3.0)
E	2014	240	201861	1.2 (1.0-1.3)
F	2014	1248	533070	2.3 (2.2-2.5)
G	2014	2275	654868	3.5 (3.3-3.6)

© 2024 Grosvenor LP et al. *JAMA Network Open.*

H	2014	2969	620151	4.8 (4.6-5.0)
I	2014	74	91282	0.8 (0.6-1.0)
J	2014	508	229034	2.2 (2.0-2.4)
K	2014	1108	294994	3.8 (3.5-4.0)
L	2014	826	283081	2.9 (2.7-3.1)
A	2015	1216	504019	2.4 (2.3-2.6)
B	2015	1811	472028	3.8 (3.7-4.0)
C	2015	11610	3410764	3.4 (3.3-3.5)
D	2015	11732	3634943	3.2 (3.2-3.3)
E	2015	303	215100	1.4 (1.3-1.6)
F	2015	1362	560342	2.4 (2.3-2.6)
G	2015	2598	718457	3.6 (3.5-3.8)
H	2015	3110	634586	4.9 (4.7-5.1)
I	2015	77	91336	0.8 (0.7-1.1)
J	2015	617	246574	2.5 (2.3-2.7)
K	2015	1201	299616	4.0 (3.8-4.2)
L	2015	880	302120	2.9 (2.7-3.1)
A	2016	1450	557027	2.6 (2.5-2.7)
B	2016	2057	485427	4.2 (4.1-4.4)
C	2016	13895	3564424	3.9 (3.8-4.0)
D	2016	13761	3814615	3.6 (3.5-3.7)
E	2016	436	219429	2.0 (1.8-2.2)
F	2016	1508	600148	2.5 (2.4-2.6)
G	2016	2703	730280	3.7 (3.6-3.8)
H	2016	3290	658608	5.0 (4.8-5.2)
I	2016	93	87532	1.1 (0.9-1.3)
J	2016	621	261074	2.4 (2.2-2.6)
K	2016	1342	311578	4.3 (4.1-4.5)
L	2016	1007	332036	3.0 (2.9-3.2)
A	2017	1712	600072	2.9 (2.7-3.0)
B	2017	2364	513591	4.6 (4.4-4.8)
C	2017	16047	3712621	4.3 (4.3-4.4)
D	2017	15812	3963655	4.0 (3.9-4.1)
E	2017	491	220010	2.2 (2.0-2.4)
F	2017	1714	602048	2.8 (2.7-3.0)
G	2017	3135	748757	4.2 (4.0-4.3)
H	2017	3076	588696	5.2 (5.0-5.4)
I	2017	104	86752	1.2 (1.0-1.5)
J	2017	726	281682	2.6 (2.4-2.8)
K	2017	1507	317525	4.7 (4.5-5.0)
L	2017	1179	331941	3.6 (3.4-3.8)
A	2018	1762	625727	2.8 (2.7-3.0)
B	2018	2805	540451	5.2 (5.0-5.4)
C	2018	18229	3859652	4.7 (4.7-4.8)
D	2018	17776	4094046	4.3 (4.3-4.4)
E	2018	574	220323	2.6 (2.4-2.8)
F	2018	1976	589288	3.4 (3.2-3.5)

© 2024 Grosvenor LP et al. *JAMA Network Open.*

G	2018	4109	819610	5.0 (4.9-5.2)
H	2018	3024	532124	5.7 (5.5-5.9)
I	2018	140	69586	2.0 (1.7-2.4)
J	2018	883	323751	2.7 (2.6-2.9)
K	2018	1580	319462	4.9 (4.7-5.2)
L	2018	1347	364034	3.7 (3.5-3.9)
A	2019	1853	617758	3.0 (2.9-3.1)
B	2019	3342	556227	6.0 (5.8-6.2)
C	2019	20501	3967940	5.2 (5.1-5.2)
D	2019	19882	4167739	4.8 (4.7-4.8)
E	2019	664	219917	3.0 (2.8-3.3)
F	2019	2294	574678	4.0 (3.8-4.2)
G	2019	4265	739187	5.8 (5.6-5.9)
H	2019	2324	409652	5.7 (5.4-5.9)
I	2019	178	68684	2.6 (2.2-3.0)
J	2019	943	285146	3.3 (3.1-3.5)
K	2019	1767	316776	5.6 (5.3-5.8)
L	2019	1338	365143	3.7 (3.5-3.9)
A	2020	1853	630075	2.9 (2.8-3.1)
B	2020	3905	569599	6.9 (6.6-7.1)
C	2020	21079	4098903	5.1 (5.1-5.2)
D	2020	20411	4269056	4.8 (4.7-4.8)
E	2020	741	222197	3.3 (3.1-3.6)
F	2020	2197	554308	4.0 (3.8-4.1)
G	2020	4432	734183	6.0 (5.9-6.2)
H	2020	2255	414803	5.4 (5.2-5.7)
I	2020	177	72513	2.4 (2.1-2.8)
J	2020	796	279523	2.8 (2.7-3.1)
K	2020	1678	325305	5.2 (4.9-5.4)
L	2020	1234	340236	3.6 (3.4-3.8)
A	2021	2199	611281	3.6 (3.5-3.8)
B	2021	4353	569305	7.6 (7.4-7.9)
C	2021	24342	4056502	6.0 (5.9-6.1)
D	2021	24184	4277211	5.7 (5.6-5.7)
E	2021	777	215298	3.6 (3.4-3.9)
F	2021	2540	527578	4.8 (4.6-5.0)
G	2021	5183	724933	7.1 (7.0-7.3)
H	2021	2128	368287	5.8 (5.5-6.0)
I	2021	223	78269	2.8 (2.5-3.2)
J	2021	955	284820	3.4 (3.1-3.6)
K	2021	1906	348742	5.5 (5.2-5.7)
L	2021	1398	339340	4.1 (3.9-4.3)
A	2022	2574	591499	4.4 (4.2-4.5)
B	2022	4913	570835	8.6 (8.4-8.8)
C	2022	23004	3849007	6.0 (5.9-6.1)
D	2022	29771	4324516	6.9 (6.8-7.0)
E	2022	935	230675	4.1 (3.8-4.3)

© 2024 Grosvenor LP et al. *JAMA Network Open.*

F	2022	2987	509756	5.9 (5.7-6.1)
G	2022	6102	733395	8.3 (8.1-8.5)
H	2022	2209	332134	6.7 (6.4-6.9)
I	2022	253	82413	3.1 (2.7-3.5)
J	2022	1076	291072	3.7 (3.5-3.9)
K	2022	2129	386664	5.5 (5.3-5.7)
L	2022	1730	362038	4.8 (4.6-5.0)

© 2024 Grosvenor LP et al. *JAMA Network Open.*

eTable 5. Count of autistic individuals and total individuals enrolled for female and male gender and each study year, with diagnosis rate per 1,000 and 95% confidence intervals (LCI = lower confidence interval, UCI = upper confidence interval). Results are shown for enrollees of all ages as well as separately for Children (0-17 years) and Adults (18+ years).

Group	Gender	Year	Autistic Individuals	Total Enrolled	Diagnosis Rate per 1000 (95% CI)
Children	Female	2011	3177	1089897	2.9 (2.8-3.0)
Children	Male	2011	14207	1139180	12.5 (12.3-12.7)
Children	Female	2012	3463	1104446	3.1 (3.0-3.2)
Children	Male	2012	15783	1157963	13.6 (13.4-13.8)
Children	Female	2013	3978	1119430	3.6 (3.4-3.7)
Children	Male	2013	17712	1168215	15.2 (14.9-15.4)
Children	Female	2014	4644	1128454	4.1 (4.0-4.2)
Children	Male	2014	20094	1179270	17.0 (16.8-17.3)
Children	Female	2015	5348	1164612	4.6 (4.5-4.7)
Children	Male	2015	22546	1217735	18.5 (18.3-18.8)
Children	Female	2016	6136	1201127	5.1 (5.0-5.2)
Children	Male	2016	25632	1254039	20.4 (20.2-20.7)
Children	Female	2017	7044	1205215	5.8 (5.7-6.0)
Children	Male	2017	28550	1258103	22.7 (22.4-23.0)
Children	Female	2018	8134	1235748	6.6 (6.4-6.7)
Children	Male	2018	32041	1291922	24.8 (24.5-25.1)
Children	Female	2019	9062	1221674	7.4 (7.3-7.6)
Children	Male	2019	34722	1276043	27.2 (26.9-27.5)
Children	Female	2020	9714	1238912	7.8 (7.7-8.0)
Children	Male	2020	35012	1293605	27.1 (26.8-27.3)
Children	Female	2021	11746	1208596	9.7 (9.5-9.9)
Children	Male	2021	39733	1261046	31.5 (31.2-31.8)
Children	Female	2022	13655	1154430	11.8 (11.6-12.0)
Children	Male	2022	42887	1204572	35.6 (35.3-35.9)
Adults	Female	2011	1064	3621146	0.3 (0.3-0.3)
Adults	Male	2011	3317	3325909	1.0 (1.0-1.0)
Adults	Female	2012	1326	3754651	0.4 (0.3-0.4)
Adults	Male	2012	3944	3437599	1.1 (1.1-1.2)
Adults	Female	2013	1458	4032297	0.4 (0.3-0.4)
Adults	Male	2013	4727	3555482	1.3 (1.3-1.4)
Adults	Female	2014	1802	4238534	0.4 (0.4-0.4)
Adults	Male	2014	5537	3756148	1.5 (1.4-1.5)
Adults	Female	2015	2118	4471271	0.5 (0.5-0.5)
Adults	Male	2015	6499	4032238	1.6 (1.6-1.7)
Adults	Female	2016	2526	4736143	0.5 (0.5-0.6)
Adults	Male	2016	7863	4260304	1.8 (1.8-1.9)
Adults	Female	2017	3025	4953525	0.6 (0.6-0.6)
Adults	Male	2017	9239	4441336	2.1 (2.0-2.1)
Adults	Female	2018	3536	5135755	0.7 (0.7-0.7)
Adults	Male	2018	10480	4606724	2.3 (2.2-2.3)
Adults	Female	2019	3979	5155301	0.8 (0.7-0.8)
Adults	Male	2019	11575	4586728	2.5 (2.5-2.6)
Adults	Female	2020	4316	5222201	0.8 (0.8-0.9)
Adults	Male	2020	11700	4672551	2.5 (2.5-2.5)
Adults	Female	2021	5158	5223666	1.0 (1.0-1.0)

© 2024 Grosvenor LP et al. *JAMA Network Open.*

Adults	Male	2021	13528	4646567	2.9 (2.9-3.0)
Adults	Female	2022	6364	5209845	1.2 (1.2-1.3)
Adults	Male	2022	14749	4657329	3.2 (3.1-3.2)

© 2024 Grosvenor LP et al. *JAMA Network Open.*

eTable 6. Male-to-female ASD prevalence ratios for each study year in 2011-2022, for the full study sample and separately for children (ages 0-17) and adults (ages 18+).

	Male-to-Female Prevalence Ratio	
**Year**	**Children**	**Adults**
2011	4.29	3.45
2012	4.34	3.29
2013	4.27	3.69
2014	4.14	3.42
2015	4.03	3.43
2016	4.00	3.49
2017	3.89	3.41
2018	3.77	3.29
2019	3.67	3.27
2020	3.45	3.01
2021	3.24	2.94
2022	3.01	2.60

© 2024 Grosvenor LP et al. *JAMA Network Open.*

eTable 7. Count of autistic individuals and total individuals enrolled each age group and each study year, with ASD diagnosis rates per 1,000 and 95% confidence intervals.

Age Group	Year	Autistic Individuals	Total Enrolled	Diagnosis Rate per 1000 (95% CI)
0-4	2011	3586	562018	6.4 (6.2-6.6)
5-8	2011	4602	466258	9.9 (9.6-10.2)
9-12	2011	4595	506600	9.1 (8.8-9.3)
13-17	2011	4603	694436	6.6 (6.4-6.8)
18-25	2011	2808	926586	3.0 (2.9-3.1)
26-34	2011	673	1002774	0.7 (0.6-0.7)
35-44	2011	381	1283498	0.3 (0.3-0.3)
45-54	2011	293	1481700	0.2 (0.2-0.2)
55-64	2011	151	1321713	0.1 (0.1-0.1)
65+	2011	76	1155061	0.1 (0.1-0.1)
0-4	2012	3959	574439	6.9 (6.7-7.1)
5-8	2012	5073	479051	10.6 (10.3-10.9)
9-12	2012	5077	511132	9.9 (9.7-10.2)
13-17	2012	5140	702843	7.3 (7.1-7.5)
18-25	2012	3334	988407	3.4 (3.3-3.5)
26-34	2012	851	1034596	0.8 (0.8-0.9)
35-44	2012	432	1303429	0.3 (0.3-0.4)
45-54	2012	345	1499997	0.2 (0.2-0.3)
55-64	2012	194	1364109	0.1 (0.1-0.2)
65+	2012	116	1215769	0.1 (0.1-0.1)
0-4	2013	4737	578209	8.2 (8.0-8.4)
5-8	2013	5636	488370	11.5 (11.2-11.8)
9-12	2013	5646	515680	10.9 (10.7-11.2)
13-17	2013	5674	705635	8.0 (7.8-8.3)
18-25	2013	3982	1026865	3.9 (3.8-4.0)
26-34	2013	1002	1056034	0.9 (0.9-1.0)
35-44	2013	505	1329800	0.4 (0.3-0.4)
45-54	2013	371	1507289	0.2 (0.2-0.3)
55-64	2013	216	1397495	0.2 (0.1-0.2)
65+	2013	111	1450811	0.1 (0.1-0.1)
0-4	2014	5393	584749	9.2 (9.0-9.5)
5-8	2014	6495	496587	13.1 (12.8-13.4)
9-12	2014	6328	518308	12.2 (11.9-12.5)
13-17	2014	6527	708299	9.2 (9.0-9.4)
18-25	2014	4658	1070075	4.4 (4.2-4.5)
26-34	2014	1283	1119180	1.1 (1.1-1.2)
35-44	2014	565	1378814	0.4 (0.4-0.4)
45-54	2014	450	1540171	0.3 (0.3-0.3)
55-64	2014	248	1454048	0.2 (0.2-0.2)
65+	2014	137	1509449	0.1 (0.1-0.1)
0-4	2015	6307	606129	10.4 (10.2-10.7)
5-8	2015	7279	512711	14.2 (13.9-14.5)
9-12	2015	7091	537449	13.2 (12.9-13.5)
13-17	2015	7221	728649	9.9 (9.7-10.1)
18-25	2015	5377	1141858	4.7 (4.6-4.8)
26-34	2015	1591	1238868	1.3 (1.2-1.3)
35-44	2015	676	1461128	0.5 (0.4-0.5)
45-54	2015	513	1629988	0.3 (0.3-0.3)
55-64	2015	295	1553768	0.2 (0.2-0.2)

© 2024 Grosvenor LP et al. *JAMA Network Open.*

65+	2015	167	1599450	0.1 (0.1-0.1)
0-4	2016	7411	631460	11.7 (11.5-12.0)
5-8	2016	8454	523120	16.2 (15.8-16.5)
9-12	2016	7759	557277	13.9 (13.6-14.2)
13-17	2016	8148	743522	11.0 (10.7-11.2)
18-25	2016	6461	1186034	5.4 (5.3-5.6)
26-34	2016	2054	1333099	1.5 (1.5-1.6)
35-44	2016	798	1534753	0.5 (0.5-0.6)
45-54	2016	552	1680510	0.3 (0.3-0.4)
55-64	2016	340	1641294	0.2 (0.2-0.2)
65+	2016	186	1697435	0.1 (0.1-0.1)
0-4	2017	8621	642003	13.4 (13.1-13.7)
5-8	2017	9406	521309	18.0 (17.7-18.4)
9-12	2017	8494	562624	15.1 (14.8-15.4)
13-17	2017	9080	737603	12.3 (12.1-12.6)
18-25	2017	7424	1202903	6.2 (6.0-6.3)
26-34	2017	2630	1413635	1.9 (1.8-1.9)
35-44	2017	935	1604007	0.6 (0.5-0.6)
45-54	2017	615	1726039	0.4 (0.3-0.4)
55-64	2017	414	1700943	0.2 (0.2-0.3)
65+	2017	248	1804788	0.1 (0.1-0.2)
0-4	2018	10147	663293	15.3 (15.0-15.6)
5-8	2018	10632	533191	19.9 (19.6-20.3)
9-12	2018	9315	580557	16.0 (15.7-16.4)
13-17	2018	10089	752077	13.4 (13.2-13.7)
18-25	2018	8255	1222136	6.8 (6.6-6.9)
26-34	2018	3209	1488613	2.2 (2.1-2.2)
35-44	2018	1097	1681513	0.7 (0.6-0.7)
45-54	2018	679	1756788	0.4 (0.4-0.4)
55-64	2018	495	1766654	0.3 (0.3-0.3)
65+	2018	287	1902045	0.2 (0.1-0.2)
0-4	2019	11758	656378	17.9 (17.6-18.2)
5-8	2019	11609	527321	22.0 (21.6-22.4)
9-12	2019	9944	575671	17.3 (16.9-17.6)
13-17	2019	10481	738586	14.2 (13.9-14.5)
18-25	2019	8925	1198513	7.4 (7.3-7.6)
26-34	2019	3745	1491960	2.5 (2.4-2.6)
35-44	2019	1324	1693331	0.8 (0.7-0.8)
45-54	2019	736	1707880	0.4 (0.4-0.5)
55-64	2019	521	1736796	0.3 (0.3-0.3)
65+	2019	308	1951253	0.2 (0.1-0.2)
0-4	2020	12470	660726	18.9 (18.5-19.2)
5-8	2020	12184	537535	22.7 (22.3-23.1)
9-12	2020	9847	578827	17.0 (16.7-17.3)
13-17	2020	10234	755686	13.5 (13.3-13.8)
18-25	2020	8874	1221164	7.3 (7.1-7.4)
26-34	2020	3995	1536935	2.6 (2.5-2.7)
35-44	2020	1491	1748144	0.9 (0.8-0.9)
45-54	2020	726	1714298	0.4 (0.4-0.5)
55-64	2020	565	1756292	0.3 (0.3-0.3)
65+	2020	372	1983977	0.2 (0.2-0.2)
0-4	2021	15306	633881	24.1 (23.8-24.5)
5-8	2021	13652	525388	26.0 (25.6-26.4)

© 2024 Grosvenor LP et al. *JAMA Network Open.*

9-12	2021	11010	558989	19.7 (19.3-20.1)
13-17	2021	11521	751662	15.3 (15.1-15.6)
18-25	2021	10124	1199256	8.4 (8.3-8.6)
26-34	2021	4877	1507305	3.2 (3.1-3.3)
35-44	2021	1820	1748066	1.0 (1.0-1.1)
45-54	2021	853	1687675	0.5 (0.5-0.5)
55-64	2021	603	1721077	0.4 (0.3-0.4)
65+	2021	422	2055916	0.2 (0.2-0.2)
0-4	2022	17315	601042	28.8 (28.4-29.2)
5-8	2022	15211	502298	30.3 (29.8-30.8)
9-12	2022	11698	530393	22.1 (21.7-22.5)
13-17	2022	12329	725626	17.0 (16.7-17.3)
18-25	2022	11185	1173625	9.5 (9.4-9.7)
26-34	2022	5504	1485262	3.7 (3.6-3.8)
35-44	2022	2276	1751364	1.3 (1.2-1.4)
45-54	2022	1006	1672683	0.6 (0.6-0.6)
55-64	2022	665	1704694	0.4 (0.4-0.4)
65+	2022	494	2116993	0.2 (0.2-0.3)

© 2024 Grosvenor LP et al. *JAMA Network Open.*

eTable 8. Count of autistic individuals and total individuals enrolled for children and adults with diagnosis rate per 1,000 and 95% confidence intervals, stratified by race group for each year in 2011-2022 (AS = Asian, BA = Black/African American, HP = Hawaiian Pacific, IN = Native American/Alaskan Native, OT = Other, WH = White).

Group	Race	Year	Autistic Individuals	Total Enrolled	Diagnosis Rate per 1,000 (95% CI)
Children	AS	2011	1950	265645	7.3 (7.0-7.7)
Children	BA	2011	1366	210343	6.5 (6.2-6.8)
Children	HP	2011	146	40289	3.6 (3.1-4.3)
Children	IN	2011	146	14637	10.0 (8.5-11.7)
Children	OT	2011	85	12830	6.6 (5.4-8.2)
Children	WH	2011	10329	1110160	9.3 (9.1-9.5)
Children	AS	2012	2270	276711	8.2 (7.9-8.5)
Children	BA	2012	1585	211772	7.5 (7.1-7.9)
Children	HP	2012	163	39975	4.1 (3.5-4.8)
Children	IN	2012	163	17041	9.6 (8.2-11.1)
Children	OT	2012	117	14378	8.1 (6.8-9.7)
Children	WH	2012	11349	1144089	9.9 (9.7-10.1)
Children	AS	2013	2728	285176	9.6 (9.2-9.9)
Children	BA	2013	1790	212362	8.4 (8.0-8.8)
Children	HP	2013	205	39978	5.1 (4.5-5.9)
Children	IN	2013	188	16757	11.2 (9.7-12.9)
Children	OT	2013	165	16567	10.0 (8.6-11.6)
Children	WH	2013	12442	1156917	10.8 (10.6-10.9)
Children	AS	2014	3345	291771	11.5 (11.1-11.9)
Children	BA	2014	2044	211759	9.7 (9.2-10.1)
Children	HP	2014	251	40406	6.2 (5.5-7.0)
Children	IN	2014	231	16704	13.8 (12.2-15.7)
Children	OT	2014	184	18835	9.8 (8.5-11.3)
Children	WH	2014	13934	1162521	12.0 (11.8-12.2)
Children	AS	2015	3960	305672	13.0 (12.6-13.4)
Children	BA	2015	2383	214437	11.1 (10.7-11.6)
Children	HP	2015	296	41574	7.1 (6.4-8.0)
Children	IN	2015	268	17497	15.3 (13.6-17.2)
Children	OT	2015	193	18755	10.3 (8.9-11.8)
Children	WH	2015	15234	1193177	12.8 (12.6-13.0)
Children	AS	2016	4718	319266	14.8 (14.4-15.2)
Children	BA	2016	2679	211618	12.7 (12.2-13.1)
Children	HP	2016	383	42554	9.0 (8.1-9.9)
Children	IN	2016	294	17669	16.6 (14.9-18.6)
Children	OT	2016	237	19945	11.9 (10.5-13.5)
Children	WH	2016	16898	1223812	13.8 (13.6-14.0)
Children	AS	2017	5468	331332	16.5 (16.1-16.9)
Children	BA	2017	3045	210415	14.5 (14.0-15.0)
Children	HP	2017	440	42669	10.3 (9.4-11.3)
Children	IN	2017	318	17639	18.0 (16.2-20.1)
Children	OT	2017	243	20262	12.0 (10.6-13.6)
Children	WH	2017	18589	1228681	15.1 (14.9-15.3)
Children	AS	2018	6287	347073	18.1 (17.7-18.6)
Children	BA	2018	3528	219759	16.1 (15.5-16.6)
Children	HP	2018	480	43567	11.0 (10.1-12.0)
Children	IN	2018	337	19184	17.6 (15.8-19.5)

© 2024 Grosvenor LP et al. *JAMA Network Open.*

Children	OT	2018	314	22963	13.7 (12.3-15.3)
Children	WH	2018	20679	1247573	16.6 (16.4-16.8)
Children	AS	2019	7078	352625	20.1 (19.6-20.5)
Children	BA	2019	3901	217148	18.0 (17.4-18.5)
Children	HP	2019	559	43960	12.7 (11.7-13.8)
Children	IN	2019	401	19178	20.9 (19.0-23.0)
Children	OT	2019	343	23140	14.8 (13.3-16.5)
Children	WH	2019	22133	1227066	18.0 (17.8-18.3)
Children	AS	2020	7173	358165	20.0 (19.6-20.5)
Children	BA	2020	4042	221011	18.3 (17.7-18.9)
Children	HP	2020	609	44646	13.6 (12.6-14.8)
Children	IN	2020	403	19518	20.6 (18.7-22.7)
Children	OT	2020	326	22694	14.4 (12.9-16.0)
Children	WH	2020	22313	1222800	18.2 (18.0-18.5)
Children	AS	2021	8121	349650	23.2 (22.7-23.7)
Children	BA	2021	4886	217809	22.4 (21.8-23.1)
Children	HP	2021	702	43476	16.1 (15.0-17.4)
Children	IN	2021	472	19080	24.7 (22.6-27.0)
Children	OT	2021	397	22587	17.6 (15.9-19.4)
Children	WH	2021	25076	1185444	21.2 (20.9-21.4)
Children	AS	2022	8870	339020	26.2 (25.6-26.7)
Children	BA	2022	5288	199500	26.5 (25.8-27.2)
Children	HP	2022	797	41662	19.1 (17.9-20.5)
Children	IN	2022	520	18400	28.3 (26.0-30.8)
Children	OT	2022	535	25290	21.2 (19.5-23.0)
Children	WH	2022	27457	1132072	24.3 (24.0-24.5)
Adults	AS	2011	360	596685	0.6 (0.5-0.7)
Adults	BA	2011	349	431168	0.8 (0.7-0.9)
Adults	HP	2011	29	32874	0.9 (0.6-1.3)
Adults	IN	2011	30	12393	2.4 (1.7-3.5)
Adults	OT	2011	12	13583	0.9 (0.5-1.5)
Adults	WH	2011	2943	4146652	0.7 (0.7-0.7)
Adults	AS	2012	463	706681	0.7 (0.6-0.7)
Adults	BA	2012	451	494532	0.9 (0.8-1.0)
Adults	HP	2012	27	36752	0.7 (0.5-1.1)
Adults	IN	2012	33	15377	2.1 (1.5-3.0)
Adults	OT	2012	18	19310	0.9 (0.6-1.5)
Adults	WH	2012	3519	4316436	0.8 (0.8-0.8)
Adults	AS	2013	529	674114	0.8 (0.7-0.9)
Adults	BA	2013	525	438203	1.2 (1.1-1.3)
Adults	HP	2013	41	39164	1.0 (0.8-1.4)
Adults	IN	2013	43	20464	2.1 (1.6-2.8)
Adults	OT	2013	28	23441	1.2 (0.8-1.7)
Adults	WH	2013	4117	4540529	0.9 (0.9-0.9)
Adults	AS	2014	664	744886	0.9 (0.8-1.0)
Adults	BA	2014	607	465317	1.3 (1.2-1.4)
Adults	HP	2014	42	57193	0.7 (0.5-1.0)
Adults	IN	2014	47	31922	1.5 (1.1-2.0)
Adults	OT	2014	29	21314	1.4 (0.9-2.0)
Adults	WH	2014	4892	4697175	1.0 (1.0-1.1)
Adults	AS	2015	775	977711	0.8 (0.7-0.9)
Adults	BA	2015	726	567353	1.3 (1.2-1.4)

© 2024 Grosvenor LP et al. *JAMA Network Open.*

Adults	HP	2015	58	54296	1.1 (0.8-1.4)
Adults	IN	2015	81	31453	2.6 (2.1-3.2)
Adults	OT	2015	27	25269	1.1 (0.7-1.6)
Adults	WH	2015	5708	4947368	1.2 (1.1-1.2)
Adults	AS	2016	1007	977288	1.0 (1.0-1.1)
Adults	BA	2016	908	576820	1.6 (1.5-1.7)
Adults	HP	2016	76	67053	1.1 (0.9-1.4)
Adults	IN	2016	81	34818	2.3 (1.9-2.9)
Adults	OT	2016	33	34868	0.9 (0.7-1.3)
Adults	WH	2016	6774	5136480	1.3 (1.3-1.4)
Adults	AS	2017	1151	1110390	1.0 (1.0-1.1)
Adults	BA	2017	1026	643740	1.6 (1.5-1.7)
Adults	HP	2017	85	64104	1.3 (1.1-1.6)
Adults	IN	2017	110	39586	2.8 (2.3-3.3)
Adults	OT	2017	49	49899	1.0 (0.7-1.3)
Adults	WH	2017	8142	5311339	1.5 (1.5-1.6)
Adults	AS	2018	1363	1196514	1.1 (1.1-1.2)
Adults	BA	2018	1153	655761	1.8 (1.7-1.9)
Adults	HP	2018	101	65925	1.5 (1.3-1.9)
Adults	IN	2018	121	44178	2.7 (2.3-3.3)
Adults	OT	2018	66	49462	1.3 (1.0-1.7)
Adults	WH	2018	9267	5469884	1.7 (1.7-1.7)
Adults	AS	2019	1550	1230180	1.3 (1.2-1.3)
Adults	BA	2019	1288	681482	1.9 (1.8-2.0)
Adults	HP	2019	133	74909	1.8 (1.5-2.1)
Adults	IN	2019	145	42647	3.4 (2.9-4.0)
Adults	OT	2019	74	40195	1.8 (1.5-2.3)
Adults	WH	2019	10289	5427933	1.9 (1.9-1.9)
Adults	AS	2020	1478	1279651	1.2 (1.1-1.2)
Adults	BA	2020	1320	684973	1.9 (1.8-2.0)
Adults	HP	2020	135	85120	1.6 (1.3-1.9)
Adults	IN	2020	156	47550	3.3 (2.8-3.8)
Adults	OT	2020	88	61380	1.4 (1.2-1.8)
Adults	WH	2020	10736	5459869	2.0 (1.9-2.0)
Adults	AS	2021	1780	1297937	1.4 (1.3-1.4)
Adults	BA	2021	1499	690936	2.2 (2.1-2.3)
Adults	HP	2021	148	93525	1.6 (1.3-1.9)
Adults	IN	2021	203	49531	4.1 (3.6-4.7)
Adults	OT	2021	114	61871	1.8 (1.5-2.2)
Adults	WH	2021	12502	5393356	2.3 (2.3-2.4)
Adults	AS	2022	1949	1306063	1.5 (1.4-1.6)
Adults	BA	2022	1677	710379	2.4 (2.3-2.5)
Adults	HP	2022	183	92472	2.0 (1.7-2.3)
Adults	IN	2022	237	53601	4.4 (3.9-5.0)
Adults	OT	2022	116	49444	2.3 (2.0-2.8)
Adults	WH	2022	14161	5324864	2.7 (2.6-2.7)

© 2024 Grosvenor LP et al. *JAMA Network Open.*

eTable 9. Count of autistic individuals and total individuals enrolled with ASD diagnosis rate per 1,000 and 95% confidence intervals for children and adults in each ethnicity group (Hispanic, Non-Hispanic) and each study year in 2011-2022.

Group	Ethnicity	Year	Autistic Individuals	Total Enrolled	Diagnosis Rate per 1,000 (95% CI)
Children	Hispanic	2011	4106	660928	6.2 (6.0-6.4)
Children	Non-Hispanic	2011	7154	724417	9.9 (9.7-10.1)
Children	Hispanic	2012	4651	675677	6.9 (6.7-7.1)
Children	Non-Hispanic	2012	7889	762892	10.3 (10.1-10.6)
Children	Hispanic	2013	5386	687646	7.8 (7.6-8.0)
Children	Non-Hispanic	2013	8619	776774	11.1 (10.9-11.3)
Children	Hispanic	2014	6207	688498	9.0 (8.8-9.2)
Children	Non-Hispanic	2014	9474	783656	12.1 (11.8-12.3)
Children	Hispanic	2015	7129	709840	10.0 (9.8-10.3)
Children	Non-Hispanic	2015	10215	788219	13.0 (12.7-13.2)
Children	Hispanic	2016	8338	729480	11.4 (11.2-11.7)
Children	Non-Hispanic	2016	10994	774925	14.2 (13.9-14.5)
Children	Hispanic	2017	9628	723178	13.3 (13.1-13.6)
Children	Non-Hispanic	2017	11756	751987	15.6 (15.4-15.9)
Children	Hispanic	2018	10938	733706	14.9 (14.6-15.2)
Children	Non-Hispanic	2018	12803	749802	17.1 (16.8-17.4)
Children	Hispanic	2019	12467	733910	17.0 (16.7-17.3)
Children	Non-Hispanic	2019	13340	715153	18.7 (18.3-19.0)
Children	Hispanic	2020	13005	742467	17.5 (17.2-17.8)
Children	Non-Hispanic	2020	13015	691890	18.8 (18.5-19.1)
Children	Hispanic	2021	15588	730817	21.3 (21.0-21.7)
Children	Non-Hispanic	2021	13900	657072	21.2 (20.8-21.5)
Children	Hispanic	2022	17725	688689	25.7 (25.4-26.1)
Children	Non-Hispanic	2022	15514	638067	24.3 (23.9-24.7)
Adults	Hispanic	2011	571	1282161	0.4 (0.4-0.5)
Adults	Non-Hispanic	2011	2044	2874625	0.7 (0.7-0.7)
Adults	Hispanic	2012	744	1350182	0.6 (0.5-0.6)
Adults	Non-Hispanic	2012	2514	2984965	0.8 (0.8-0.9)
Adults	Hispanic	2013	879	1402764	0.6 (0.6-0.7)
Adults	Non-Hispanic	2013	2950	3237211	0.9 (0.9-0.9)
Adults	Hispanic	2014	1084	1516751	0.7 (0.7-0.8)
Adults	Non-Hispanic	2014	3466	3319315	1.0 (1.0-1.1)
Adults	Hispanic	2015	1352	1688690	0.8 (0.8-0.8)
Adults	Non-Hispanic	2015	4037	3464060	1.2 (1.1-1.2)
Adults	Hispanic	2016	1732	1804093	1.0 (0.9-1.0)
Adults	Non-Hispanic	2016	4732	3512680	1.3 (1.3-1.4)
Adults	Hispanic	2017	2121	1910140	1.1 (1.1-1.2)
Adults	Non-Hispanic	2017	5494	3590601	1.5 (1.5-1.6)
Adults	Hispanic	2018	2476	2019367	1.2 (1.2-1.3)
Adults	Non-Hispanic	2018	6227	3691460	1.7 (1.6-1.7)
Adults	Hispanic	2019	2809	2109097	1.3 (1.3-1.4)
Adults	Non-Hispanic	2019	6800	3640290	1.9 (1.8-1.9)
Adults	Hispanic	2020	2783	2186442	1.3 (1.2-1.3)
Adults	Non-Hispanic	2020	7057	3611780	2.0 (1.9-2.0)
Adults	Hispanic	2021	3402	2220738	1.5 (1.5-1.6)
Adults	Non-Hispanic	2021	8292	3554335	2.3 (2.3-2.4)

© 2024 Grosvenor LP et al. *JAMA Network Open.*

Adults	Hispanic	2022	4050	2253599	1.8 (1.7-1.9)
Adults	Non-Hispanic	2022	9749	3549838	2.7 (2.7-2.8)

© 2024 Grosvenor LP et al. *JAMA Network Open.*

eTable 10. Results from weighted least squares models testing significance of changes in diagnosis rates from 2011-2022 within strata of Age, Gender, Race, and Ethnicity (EAPC = estimated annual percentage change; LCI = lower confidence interval; UCI = upper confidence interval).

**Age Group (Years)**	**EAPC**	**LCI**	**UCI**	***P***
0-4	15.19	14.05	16.33	<.001
5-8	10.62	9.51	11.72	<.001
9-12	7.73	6.66	8.80	<.001
13-17	8.28	7.21	9.36	<.001
18-25	10.24	9.15	11.33	<.001
26-34	16.07	14.91	17.22	<.001
35-44	15.13	14.00	16.27	<.001
45-54	9.89	8.80	10.97	<.001
55-64	10.93	9.83	12.04	<.001
65+	11.35	10.25	12.45	<.001

Gender

**Group**	**Gender**	**EAPC**	**LCI**	**UCI**	***P***
Children (0-17)	Female	13.62	12.49	14.75	<.001
Children (0-17)	Male	9.63	8.54	10.72	<.001
Adults (18+)	Female	13.74	12.61	14.86	<.001
Adults (18+)	Male	10.34	9.24	11.43	<.001

Race

**Group**	**Race**	**EAPC**	**LCI**	**UCI**	***P***
Children (0-17)	AS	11.5	10.39	12.6	<.001
Children (0-17)	BA	13.1	11.99	14.22	<.001
Children (0-17)	HP	13.95	12.82	15.08	<.001
Children (0-17)	IN	9.23	8.15	10.31	<.001
Children (0-17)	OT	8.97	7.89	10.05	<.001
Children (0-17)	WH	8.82	7.74	9.89	<.001
Adults (18+)	AS	8.28	7.21	9.35	<.001
Adults (18+)	BA	8.89	7.81	9.96	<.001
Adults (18+)	HP	9.62	8.54	10.7	<.001
Adults (18+)	IN	8.36	7.29	9.43	<.001
Adults (18+)	OT	9.18	8.1	10.26	<.001
Adults (18+)	WH	12.29	11.19	13.4	<.001

Ethnicity

**Group**	**Ethnicity**	**EAPC**	**LCI**	**UCI**	***P***
Children (0-17)	Hispanic	13.38	12.26	14.50	<.001
Children (0-17)	Non-Hispanic	8.50	7.43	9.57	<.001
Adults (18+)	Hispanic	11.89	10.78	12.99	<.001
Adults (18+)	Non-Hispanic	12.45	11.33	13.56	<.001

© 2024 Grosvenor LP et al. *JAMA Network Open.*

eFigure 1. Prevalence of ASD diagnosis per 1,000 enrollees among **A) c**hildren and **B)** adults enrolled at all MHRN sites from 2011-2022, stratified by reported gender.

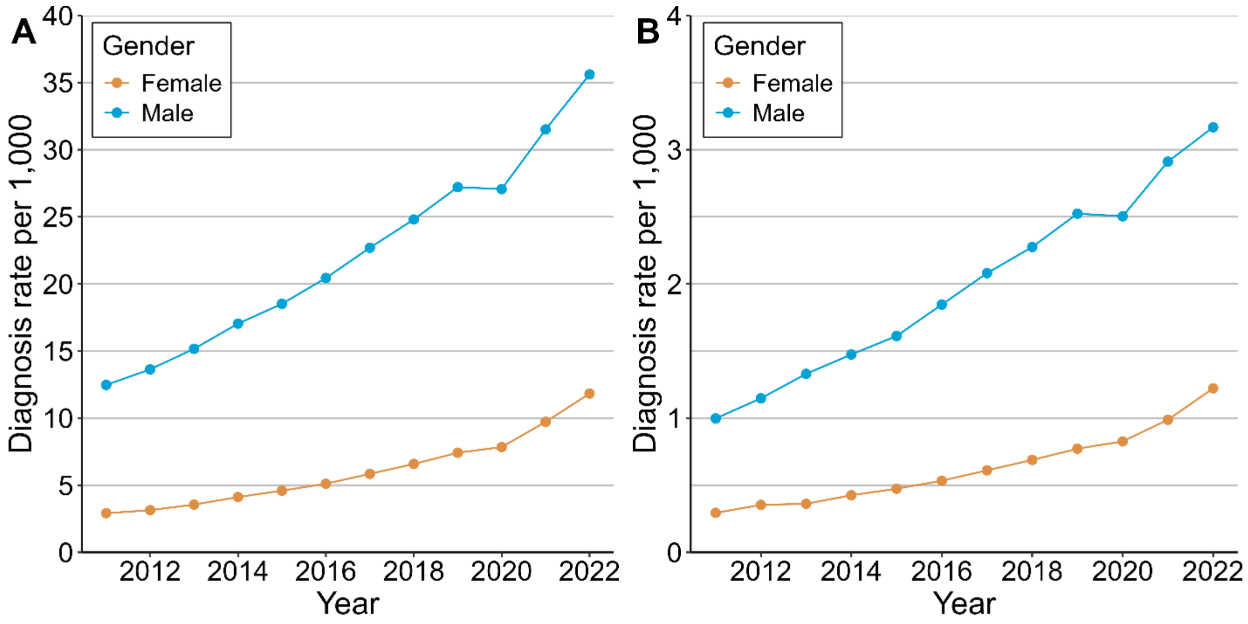

© 2024 Grosvenor LP et al. *JAMA Network Open.*

eFigure 2. Annual prevalence of ASD diagnosis per 1,000 enrollees in **A**) 2011 and **B)** 2022, stratified by age group and Hispanic ethnicity.

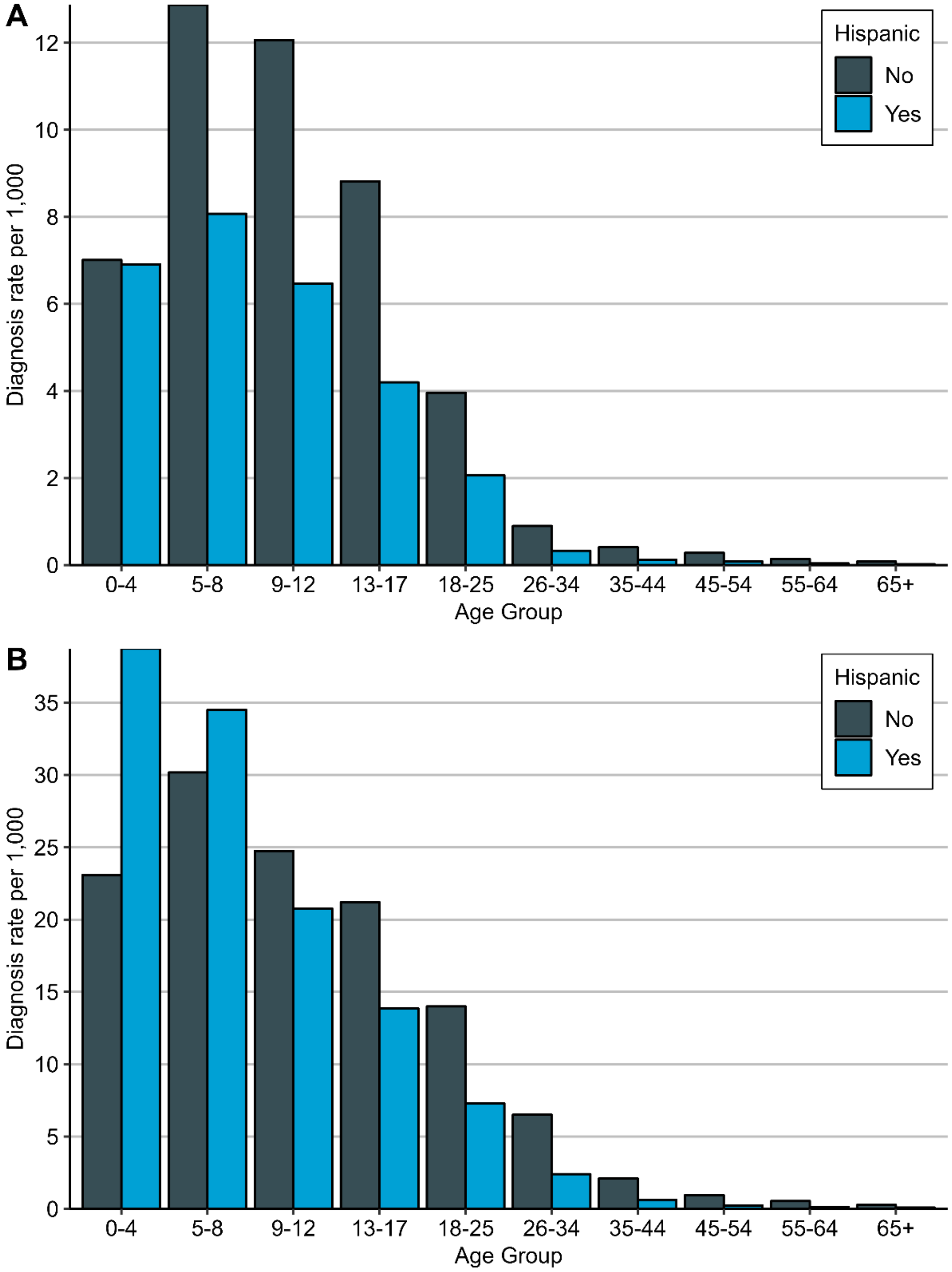

© 2024 Grosvenor LP et al. *JAMA Network Open.*
