# Supplementary material for: Autism Diagnosis Among US Children and Adults, 2011-2022
Source: JAMA Netw Open. 2024 Oct 30;7(10):e2442218. doi: 10.1001/jamanetworkopen.2024.42218 (PMC11525601; doi:10.1001/jamanetworkopen.2024.42218)
Supplement: Supplement 2. — Data Sharing Statement [file jamanetwopen-e2442218-s002.pdf]

Data Sharing Statement

Grosvenor. Autism Diagnosis Among US Children and Adults, 2011-2022. *JAMA Netw Open*. Published October 30, 2024. doi:10.1001/jamanetworkopen.2024.42218

Data
Data available: No

Additional Information
Explanation for why data not available: No individual-level patient data will be made available because only aggregate data (annual counts of diagnoses and enrolled members) were used for this study. This is also described in our Data Sharing Statement at the bottom of the manuscript.
